# Oral health and oral-health-related quality of life in people with X-linked hypophosphatemia

**DOI:** 10.1186/s12903-024-04028-9

**Published:** 2024-02-21

**Authors:** Jannik Steur, Lauren Bohner, Jochen Jackowski, Marcel Hanisch, Ole Oelerich

**Affiliations:** 1 Department of Prosthodontics, University Hospital Münster, Münster, 48149 Germany; 2 Department of Oral and Maxillofacial Surgery, University Hospital Münster, Münster, 48149 Germany; 3 Department of Oral Surgery and Policlinical Ambulance, Faculty of Health, Witten/Herdecke University, Alfred-Herrhausen-Str. 45, Witten, 58448 Germany

**Keywords:** Rare diseases, X-linked hypophosphatemia, Oral health, OHIP-14, XLH, Patient-reported outcome, PhOX

## Abstract

**Background:**

X-linked hypophosphatemia (XLH) is a type of vitamin D-resistant rickets. It is the most common form of it and is related with oral health problems. This study aimed to analyze the OHRQoL of people suffering from XLH and measure physical oral health to confirm or refute evidence of reduced oral health.

**Methods:**

The German version of the Oral Health Impact Profile (OHIP-14G), was used to measure OHRQoL. All study participants underwent clinical examination, and oral health was scored using the Physical Oral Health Index (PhOX).

**Results:**

A total of 26 people participated in the study, of whom five were male and 21 were female. The average participant age was 40.9 ± 12.8 years. The OHIP-14G score was 14.3 (± 12.1; 95% CI: 9.37. 19.16) points (range 0–44 points). The PhOX score was 77.1 (± 9.9; 95% CI: 73.10—81.13) points (range 61–95 points).

**Conclusions:**

The results of this study confirm that oral health and OHRQoL are both reduced in the studied cohort of people affected by XLH. Particular attention should be paid to perfect oral hygiene in people with XLH, as the impaired enamel mineralisation increases the risk of caries and thus also the occurrence of apical infections.

## Introduction

In the European Union, a disease is described as "rare" if less than one person in 2000 is affected [1]. X-linked hypophosphatemia (XLH) is one of them, with a prevalence of less than 1 per 20.000 people [2]. The cause of the disease is a mutation on chromosome Xp22, which causes a change in the X-linked phosphate-regulating endopeptidase (PHEX) gene [3]. XLH can vary in clinical symptoms, but bone deformities, small body size, and dental abnormalities usually characterize it. These are the consequences of hypophosphatemia and decreased mineralization of bone and teeth [4] due to decreased renal phosphate reabsorption [5].

Dental findings in XLH vary, and the exact pathogenesis remains unclear [6]. The most common oral findings are recurrent abscesses and fistulas [6–8] in both the primary and permanent dentition despite caries-free teeth. This is because, as a result of impaired mineralization of the teeth, bacteria can penetrate through the enamel and underlying dentin to reach the pulp and cause necrosis. In addition to this increased risk of pulp necrosis, people with XLH have an increased risk of periodontitis because the alveolar bone and cementum are also affected by the defective mineralisation [9, 10]. As a result of periodontal inflammation, bone loss and recession occur. These dental manifestations increase the risk of premature tooth loss [4]. Currently, there are only treatment recommendations and, due to the rarity of the disease, no universally applicable therapies to prevent, for example, apical periodontitis. Recommendations include regular dental examinations to identify and cover exposed dentin as soon as possible, sealing of fissures and pits, minimally invasive all-ceramic veneers and root canal treatment if pulp necrosis has already occurred [6]. Characteristically, the decreased dentin mineralization shows large interglobular spaces formed due to the lack of calcospherite fusion in the region close to the pulp during mineralization. Odontoblast function is not impaired, but the mineralization process of the tooth structure is affected by hypophosphatemia [11, 12]. XLH patients are significantly more likely to show enamel hypoplasia on the incisors, canines, and first molars than on the premolars or other molars, but this is not a predominant symptom [13].

The frequent occurrence of oral symptoms in XLH patients is associated with reduced OHRQoL. This has already been shown in a study by Hanisch et al. [14]. Generally, XLH sufferers show a high need for endodontic treatment due to apical periodontitis [15]. Radiologically, one can see enlarged pulp chambers that minimize the dentin and enamel layer thickness [6, 16]. In addition to the altered dentin structure and the large interglobular spaces, there is an increased risk of reinfection after endodontic treatment [17]. Another issue to consider, and one that also has a negative impact on OHRQoL, is that the dental problems caused by the disease also result in treatment costs for patients and significantly more frequent visits to the dentist, even if only for check-ups, which can also be a source of distress.

This study analyzes oral health and OHRQoL in people with XLH. This is a further step in helping those affected by highlighting existing oral problems and the associated impact on OHRQoL. This is necessary to give possible treatment recommendations for affected individuals and provide them with adequate care.

## Materials and methods

The data collection was conducted during the annual meeting of the self-help group Phosphatdiabetes e.V. in the period of May 14–15, 2022, or after a visit to the special consultation on “Rare diseases with orofacial involvement” at the Department of Cranio-Maxillofacial Surgery at the Westphalian Wilhelms University of Münster. A call for studies was sent to its members via the self-help group's e-mail distribution list, and patients with XLH were also made aware of the study during the consultation hours offered for rare diseases with oral involvement. The entire period used to collect the data ranged from October 2021 to August 2022. The clinical examination was performed by three calibrated dentists with experience in prosthodontics and/or oral surgery [J.S., O. O., M.H.]. Calibration consisted of a joint point-by-point review of the questionnaires used. In addition, prior to the start of the study, the examiners performed the objective oral health assessment using the PhOX on each other in the presence of the other examiners in order to best harmonize the data collection among the examiners.The study was approved by the Ethics Committee of the Westphalia-Lippe Dental Association and the Westphalian Wilhelms University of Münster (No. 2020–169-f-S).

### Inclusion criteria

To participate in the study, participants had to be at least 16 years old, diagnosed with XLH, and given written informed consent. Only complete questionnaires were included for the final data assessment.

### Study design

Patient information and relevant dental history were recorded (e.g., orthodontic treatment). Participants were asked to answer the German short form of the Oral Health Impact Profile (OHIP-14) [18] questionnaire. Participants were also clinically examined, and their oral health was scored using the Physical Oral Health Index (PhOX) [19].

### Assessment of oral health

A clinical dental examination was performed by one of three calibrated investigators on all study participants. The clinical examination is based on the Physical Oral Health Index (PhOX), which measures all objectively measurable oral health areas. First, information about pain or paresthesia in the oral-maxillofacial area in the past 30 days was obtained.

The condition of the teeth was assessed using a Decay-Missing-Filled Teeth (DMFT) index adapted for the PhOX (see Reissmann et al. for an explanation of the complete index [19]). The endodontium's condition was recorded by examining all teeth for pain or discomfort on percussion. The periodontium was assessed by recording the pocket probing depth of the 6 Ramfjord teeth [20]. The oral soft tissues were visually inspected. This included looking for swelling, redness, and loss of integrity. Saliva was evaluated both quantitatively (by visual assessment of moistening of the oral mucosa) and qualitatively (fluid vs foamy). The masticatory muscles and jaw (in the area of the temporalis muscle, masseter muscle with 1 kg finger pressure and temporomandibular joint, parotid gland, submandibular gland with 0.5 kg finger pressure) were palpated, and pain was rated according to intensity (mild, moderate, severe) and familiarity (known pain vs. unknown pain). Deviations from ideal tooth positions were recorded by eliciting overjet and overbite. In addition, contact point deviations > 5 mm and posterior crossbites were recorded. The normal oral function was assessed by maximum active and passive mouth opening (even when painful).

To objectify the findings, each of the 14 subitems of the PhOX was scored from 0 to 4. Depending on their relevance, the 14 subitems were weighted once, twice, or thrice. This results in an overall score that can range from 0–100. The objectively best possible oral health is assigned a value of 100.

### Assessment of oral health-related quality of life

To assess subjective oral health-related quality of life, the German short form of the Oral Health Impact Profile (OHIP-14) was used [18]. The questionnaire was distributed to participants prior to the examination for independent completion. The OHIP-14 questionnaire includes 14 questions on the frequency of pain, limitations, social or physical stress, discomfort, and difficulties related to social life. For each question, a score is given from 0 = "never", 1 = "hardly ever", 2 = "occasionally", 3 = "often" to 4 = "very often". Thus, the overall OHIP score takes on values between 0–56 points, with 56 corresponding to the worst result. For a coherent interpretation, the OHIP-14 should be assessed against the four dimensions of the OHRQoL, according to the current recommendations [21]. The four dimensions 'Oral Function', 'Orofacial Pain', 'Orofacial Appearance' and 'Psychosocial Impact' are each calculated from two of the OHIP-14 items and can therefore take values between 0–8, with 8 corresponding the worst result for the given dimension of OHRQoL.

### Statistical methods

Data were presented descriptively and graphically to represent oral health status and their perceptions of oral health quality. General participant data (i.e., age, age at diagnosis, time between onset of first symptoms to diagnosis) were analyzed descriptively.

To illustrate the differences in oral health and OHRQoL, the results of the OHIP and its four dimensions and the PhOX results were also presented in an age-dependent form. For this purpose, the participants were divided into decades. A Kruskal–Wallis test with a significance level of 0.05 was used to determine whether there were significant differences in OHIP, its four dimensions or PhOX between the different age groups.

Responses regarding the different oral conditions of the participants and their distribution are shown as percentages. For OHIP-14 and PhOX values, 95% confidence intervals (CI) are presented in addition to the maximum, minimum, mean, and standard deviation. Empirical data were analyzed using SPSS Statistics for Windows version 26.0 (IBM Corp., Armonk, NY, USA) and RStudio Version 2022.07.1 + 554 (RStudio PBC, Boston, MA, USA).

## Results

### Age and diagnosis period

A total of 26 participants were included in the study, of whom 80.8% were female, i.e., 21 individuals were female, and five individuals were male. The mean age of the participants (interquartile range, IQR) was 40.9 (22.0, range 21–63) years. The mean age at diagnosis was 10.1 (12.0) years. It took 7.4 (8.0) years from the onset of symptoms to diagnosis.

### OHRQoL

The mean OHIP-14 score was 14.3 (± 12.1, range 0–44; 95% CI: 9.37—19.16). Detailed information on each individual OHIP-Item can be seen in Table 1. The highest impairment occurred in the dimension ‘Orofacial Appearance’ (3.2 ± 2.1; 95% CI: 2.29—4.02), followed by ‘Orofacial Pain’ (2.9 ± 2.1; 95% CI: 2.06—3.79) and ‘Psychosocial Impact’ (1.6 ± 1.9; 95% CI: 0.85—2.38). The lowest impairment occurred in the dimension ‘Oral Function’ (1.5 ± 2.1; 95% CI: 0.65—2.35).

**Table 1:** Distribution of individual responses to each Oral Health Impact Profile (OHIP-14) item (%)

OHIP-14 item	Never	Hardly Ever	Occasionally	Often	Very Often
Have you had any problems with your teeth, mouth or dentures in the past month …					
… making it difficult to pronounce words?	21 (80.8)	2 (7.7)	2 (7.7)	1 (3.8)	0 (0.0)
… that make you feel that your sense of taste was impaired?	18 (69.2)	2 (7.7)	4 (15.4)	2 (7.7)	0 (0.0)
… that gave you the impression that your life in general was less satisfying?	10 (38.5)	7 (26.9)	5 (19.2)	3 (11.5)	1 (3.8)
… making it difficult to relax?	8 (30.8)	7 (26.9)	5 (19.2)	5 (19.2)	1 (3.8)
Has it often happened in the past month due to problems with your teeth, mouth or dentures …					
… that you felt tense?	8 (30.8)	3 (11.5)	7 (26.9)	8 (30.8)	0 (0.0)
… that you had to interrupt meals?	16 (61.5)	4 (15.4)	4 (15.4)	1 (3.8)	1 (3.8)
… that you were uncomfortable eating certain foods?	8 (30.8)	5 (19.2)	9 (34.6)	2 (7.7)	2 (7.7)
… that you have been irritable with other people?	16 (61.5)	3 (11.5)	4 (15.4)	3 (11.5)	0 (0.0)
… that you had difficulty doing your usual job?	15 (57.7)	5 (19.2)	3 (11.5)	3 (11.5)	0 (0.0)
… that you were completely unable to do anything?	20 (76.9)	3 (11.5)	0 (0.0)	3 (11.5)	0 (0.0)
… that you have been a bit embarrassed?	13 (50.0)	3 (11.5)	4 (15.4)	4 (15.4)	2 (7.7)
… that your diet has been unsatisfactory?	16 (61.5)	4 (15.4)	2 (7.7)	4 (15.4)	0 (0.0)
In the past month, did you have …					
… any pain in your mouth?	6 (23.1)	7 (26.9)	8 (30.8)	4 (15.4)	1 (3.8)
… a feeling of uncertainty about your teeth, mouth or dentures?	6 (23.1)	8 (30.8)	5 (19.2)	5 (19.2)	2 (7.7)

### Oral health

**Fig. 1:**
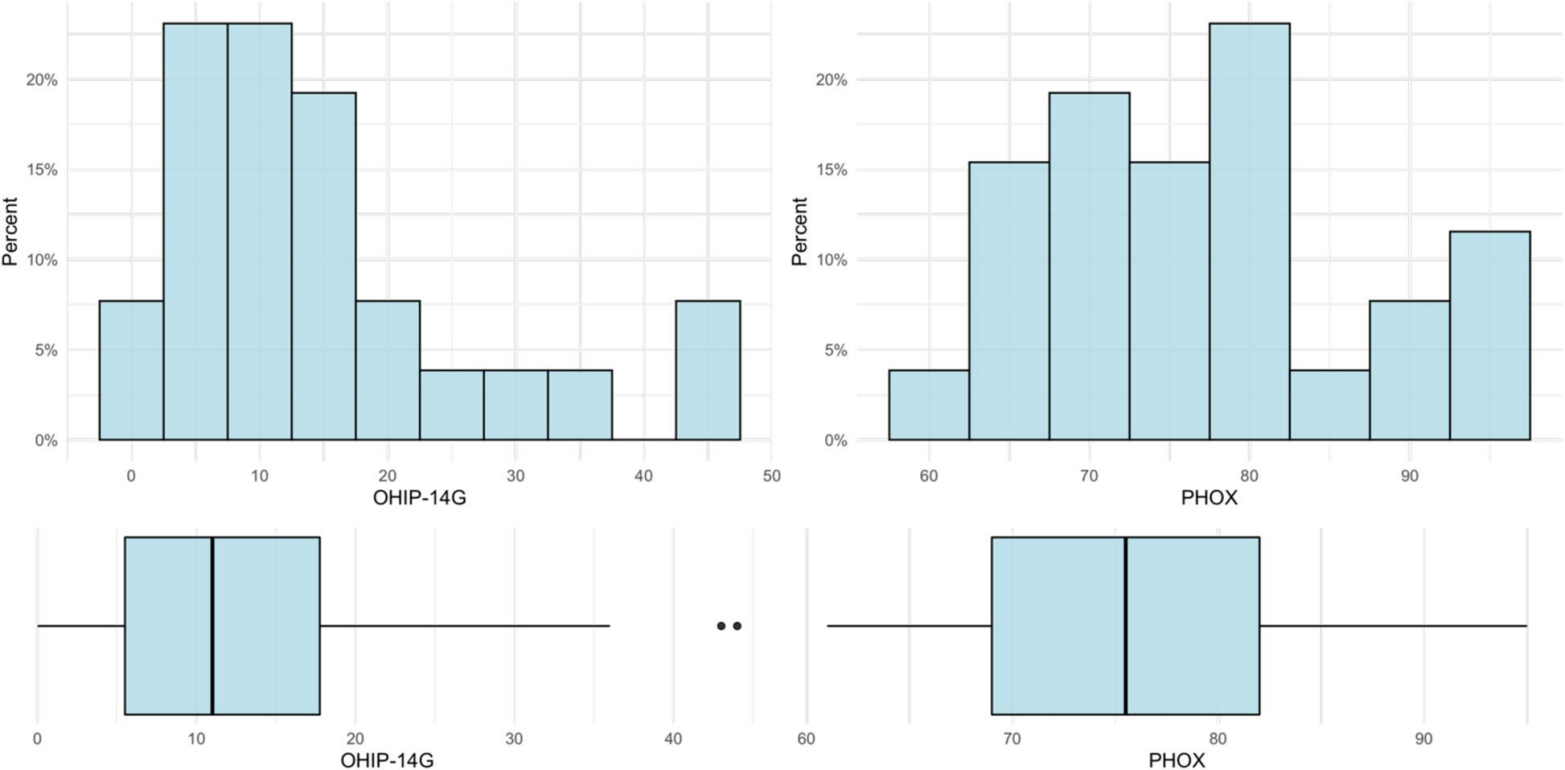
Oral health and oral health-related quality of life. Distribution of Oral Health Impact Profile (OHIP-14G) and Physical Oral Health Index (PhOX) values and corresponding box plots

All but one participant (96.2%) had residual teeth. Ten participants had complete dentition (excluding third molars) (38.5%), and only one was treated with a removable total prosthesis (3.8%). All participants with remaining teeth had at least one filling. A total of 38.5% of the participants had more filled teeth than healthy teeth but no need for treatment. A total of eight participants (30.8%) had probing depths of 3.5–5.5 mm on 1–3 teeth. Four participants (15.4%) had probing depths of 3.5–5.5 mm on more than three teeth or probing depths of more than 5.5 mm on 1–3 teeth. In addition, four participants (15.4%) had no probing depths >  = 3.5 mm and no bleeding after probing. Eleven participants (42.3%) had pain on percussion on one to seven teeth (max 25% of teeth), and 14 participants (53.8%) had no pain on percussion. One participant (3.8%) had pain on percussion on more than 50% of the teeth. Six participants (23.1%) had pain on palpation of the masticatory muscles and temporomandibular joints. Moistening of the oral area was normal in all participants. Six participants (23.1%) had mild swelling of the oral cavity, involving no more than ¼ of the total surface area. Eight participants (30.8%) reported unpleasant sensations such as burning, hypersensitivity, or numbness in the mouth several times a week. Twelve participants (46.2%) reported experiencing these uncomfortable sensations every day. Based on the diagnostic findings, the PhOX score was calculated with a mean value of 77.1 (± 9.9, range 61–95; 95% CI: 73.10—81.13). Figure 1 shows the distribution of OHIP-14 and PhOX values with corresponding box plots. Detailed information on each individual PhOX-Item can be seen in Table 2.

**Table 2:** Distribution of Physical Oral Health Index (PhOX) values per item

PhOX Values n(%)					
**Domain**	**0**	**1**	**2**	**3**	**4**
Teeth quantity	1 (3.8)	1 (3.8)	2 (7.7)	12 (46.2)	10 (38.5)
Condition of teeth	2 (7.7)	4 (15.4)	10 (38.5)	10 (38.5)	0 (0.0)
Condition of periodontium	0 (0.0)	4 (15.4)	8 (30.8)	10 (38.5)	4 (15.4)
Condition of endodontium	1 (3.8)	1 (3.8)	1 (3.8)	10 (38.5)	13 (50.0)
Surface of oral mucosa	0 (0.0)	0 (0.0)	0 (0.0)	13 (50.0)	13 (50.0)
Color of oral mucosa	0 (0.0)	6 (23.1)	0 (0.0)	7 (26.9)	13 (50.0)
Moistening of oral mucosa	0 (0.0)	0 (0.0)	1 (3.8)	0 (0.0)	25 (96.2)
Pain on palpation of jaws and muscles	0 (0.0)	0 (0.0)	6 (23.1)	0 (0.0)	20 (76.9)
Continuity of jaws, palate and tongue	0 (0.0)	0 (0.0)	0 (0.0)	0 (0.0)	26 (100)
Size ratio of jaws	0 (0.0)	0 (0.0)	2 (7.7)	5 (19.2)	19 (73.1)
Mouth opening capacity	0 (0.0)	0 (0.0)	7 (26.9)	11 (42.3)	8 (30.8)
Number of supporting zones	0 (0.0)	0 (0.0)	0 (0.0)	0 (0.0)	26 (100)
Pain frequency	0 (0.0)	1 (3.8)	5 (19.2)	8 (30.8)	12 (46.2)
Paresthesia frequency	0 (0.0)	1 (3.8)	5 (19.2)	8 (30.8)	12 (46.2)

### Age related changes in OHRQoL and oral health

**Fig. 2:**
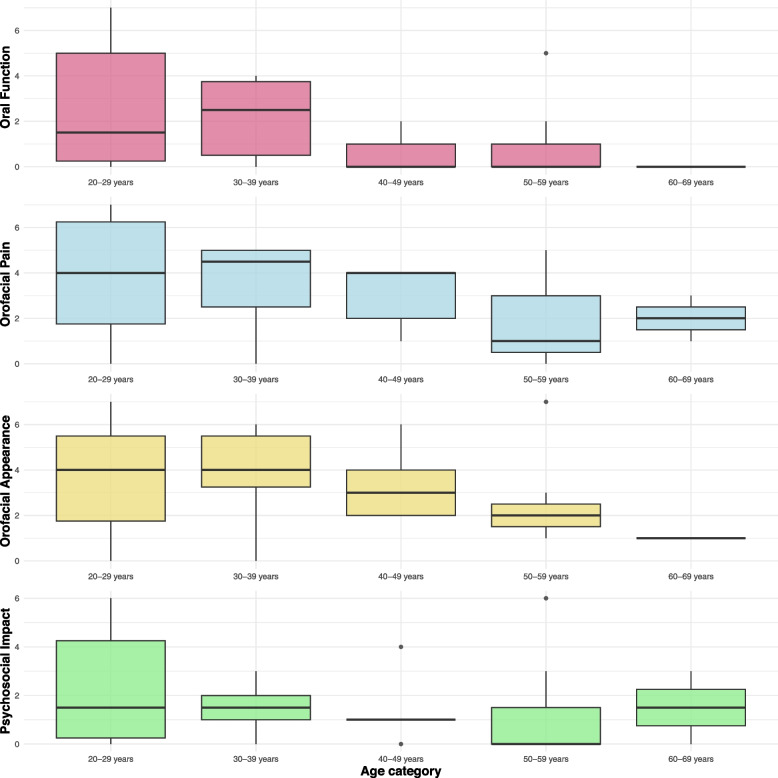
Age-related data for individual dimensions of OHRQoL. For age-dependent values, the participants were divided into decades and the four dimensions of the OHRQoL were represented by box plots

When analysing age-related OHRQoL, no significant difference was found between age groups (*p* = 0.827). The OHIP score was lowest in the 50–59 age group (median = 7) and highest in the 30–39 age group (median = 15.5). These results are also comparable for the four different dimensions of the OHRQoL. The 50–50 age group had the lowest scores in all dimensions except the Orofacial Appearance dimension. Again, there were no significant differences between the age groups (Oral Function *p* = 0.292; Orofacial Pain *p* = 0.491; Orofacial Appearance *p* = 0.375; Psychosocial Impact *p* = 0.789). Box plots for each age group and the four dimensions of the OHRQoL are shown in Fig. 2.

**Fig. 3:**
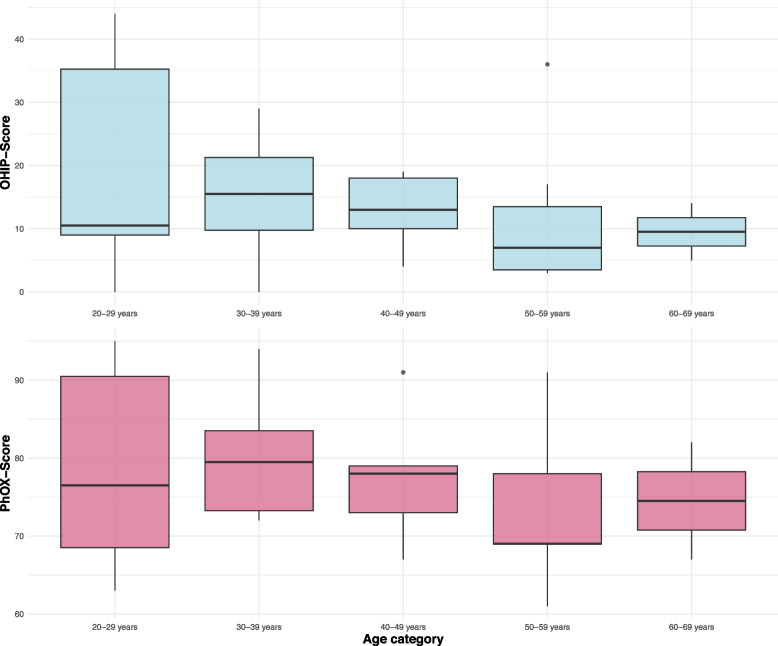
Age-related data for OHIP- and PhOX-Score. The age-dependent values are shown in decades and corresponding box plots

There was also no significant difference in oral health between age groups (PhOX *p* = 0.779). The best oral health was found in the 30–39 age group (median = 79.5), while the worst oral health was found in the 50–59 age group (median = 69). The summary scores of the OHIP and PhOX are shown in Fig. 3 as box plots in relation to the age groups.

## Discussion

This is the first study to investigate both OHRQoL and measured oral health in patients with XLH. Previous studies have described the oral symptoms associated with XLH and found that overall oral health, but also OHRQoL, is reduced as a result. Our study attempts to establish a relationship between these two factors in order to map objective oral health and subjective OHRQoL in a single cohort. Although only 26 participants affected by this rare disease could participate in this study, the results show that both OHRQoL and measured oral health were reduced. These results are consistent with previous studies showing that the objective and subjective oral health of people with XLH is more impaired compared to the general German population [19, 22].

Our study shows a clear imbalance between the two sexes. More than 80% of the participants in our cohort were female. This is higher than the male-to-female ratio of 1–1.5 to 1–2 reported in other studies [23, 24]. One reason for this discrepancy may be that we did not have a central registry to rely on for recruitment, but relied on patient support groups. Previous studies of rare diseases with orofacial involvement that used patient support groups also had higher proportions of female participants, which could indicate that women are either more represented in patient support groups or more willing to participate in voluntary studies [14, 25, 26].

Almost half of the patients (twelve) had at least one percussion-sensitive tooth. Percussion-sensitive teeth may indicate pathological apical changes. In addition, many patients already had several extensive, and in most cases sufficient, fillings. Previous studies have shown that individuals with XLH have numerous histopathological structural abnormalities in deciduous and permanent teeth, as evidenced by a lack of calcosphere fusion in interglobular dentin and unmineralized dentin. As a result, even minor lesions can expose the altered dentin, facilitating bacterial invasion and infection of dental tissues [17, 27, 28]. This dentin cannot adequately protect the pulp chamber, and bacteria can cause pulpitis. The increased need for endodontic treatment increases proportionally with patient age, showing that apical periodontitis correlates with abrasion and attrition [29]. For this reason we have chosen to present the results for OHRQoL and Oral Health as a function of age. Although there were no significant differences between the age groups for both OHIP and PhOX, the results give rise to discussion. On the one hand, people in the 50–59 age group have the lowest PhOX scores and therefore the lowest oral health scores. On the other hand, this group of people reported the lowest restriction of OHRQoL. These results cannot be explained at present and may be related to the small number of participants. Therefore, larger case–control studies should be conducted to find possible explanations for our results, as it can be generally assumed that dental problems become more frequent with increasing age in XLH [4], and thus a negative influence on OHRQoL could also be expected.

As apical periodontitis is one of the main and known problems in people with XLH, the most crucial aspect is optimal oral hygiene and regular dental check-ups. The idea is that exposed dentin provides an easier entry point for bacterial infection of the teeths [6, 30]. If these areas are identified as early as possible through regular check-ups, measures can be taken to stop or at least slow down the progression of the lesion. As there is a high risk of undermining defects and pulp necrosis even with visibly intact enamel and good oral hygiene, check-ups and perfect oral hygiene can only be a supporting factor [4]. For this reason, routine x-rays are extremely important for people with XLH. These X-rays should be taken at regular intervals, even if there are no visible carious lesions, as this is not a reliable indicator in affected individuals, as described above [30, 31]. As we only assessed possible endodontic problems by percussion in our study, our results should be verified by radiographs in future studies.

There are different treatment approaches to prophylaxis. These include crowning and sealing the teeth with flowable composite, as Sabandal et al. [6] and Rathore et al. [16] already described. Although there are no valid scientific data on the treatment options, it can be said that the need to prepare teeth for crowns and the associated removal or reduction of intact enamel increases the risk of further microcracks developing in the tooth substance, through which the altered dentin can be reinfected [30]. Therefore, the benefits of minimally invasive tooth sealing outweigh the risks. A suggested option for covering larger areas affected by attrition in a substance-preserving manner is the use of all-ceramic occlusal veneers [6].

None of the participants had a relative or absolute dry mouth on clinical examination, and saliva consistency was also physiological in 25 of the 26 participants. The factors of reduced salivary flow and altered salivary consistency, which have a negative impact on tooth structure due to reduced remineralization, can be considered incidental to the cause of the need for treatment and oral manifestations in people with XLH, as our findings provide no evidence that remineralization is not achieved by saliva [32].

A strikingly high proportion of participants (73.1%) had received orthodontic treatment. Altered bone mineralization and the development of bone deformities in XLH patients could be a possible reason for the increased need for orthodontic treatment. There have been no studies of orthodontic treatment in XLH patients. However, a study by Dumbryte et al. shows that removing brackets increases the size of preexisting microcracks in the enamel and causes new microcracks to form [33]. The resulting or enlarged microcracks are not a problem in healthy patients. In XLH patients however, this facilitates the penetration of bacteria into the tooth structure due to the altered dentin. To prevent this, orthodontic treatment in XLH patients must be further investigated and reconsidered if necessary. A first approach could be to treat particular patients mainly with aligners, as tooth movements are possible here without attachment to the tooth. In this way, the tooth surface remains untouched, and no new or enlarged microcracks are created.

The dental findings in the individuals with XLH in our study could potentially lead to a decrease of OHRQoL. The mean OHIP-14G score in this study was 14.3 (95% CI: 9.37—19.16). Similar results were found in a previous study of XLH, with an OHIP-14G score of 10.3 [14]. Compared to the average OHIP score of the German population (4.09), this indicates impaired OHRQoL in the study participants [34]. To better interpret the influence of one point of the OHIP, Reissmann et al. measured that one point represents approximately 15.2 impairments per month in the extended version of the OHIP (OHIP-49). The authors suggest that this relates to 53.2 impacts per month for each point of the short form OHIP-14 [35].

Only two of the 26 participants had the minimum possible score of 0 points (no impact of oral health on quality of life), and none reached the maximum score of 56 points (very high impact of oral health on quality of life). Oral health had the lowest impact on OHRQoL regarding the "ability to pronounce words correctly" and "complete inability to do anything." In contrast, oral health had the highest impact on "uncertainty," "pain," and "tension".

Dental findings and the diagnostic process in XLH can negatively influence OHRQoL. The mean time between the onset of the first symptoms to diagnosis in our study was 7.4 years. Similar results were found in a study by Bohner et al., which found that OHRQoL steadily worsened with a delay in diagnosis in a cohort of 473 individuals affected by a rare disease, with an average time of diagnosis of 8.37 years after the onset of the first symptoms [36]. Another study showed comparable results (an average of 7 years for people affected by a rare disease) [37]. In our cohort, however, the participants with the most prolonged delay in diagnosis did not show the worst OHIP-14 score. XLH disease in participants with the highest OHIP scores (44 and 43) was detected at birth. As already mentioned, OHIP-14 scores of 0 were also obtained twice, in which the diagnosis was also made immediately at birth and one year after the onset of the first symptoms.

A study conducted by Hanisch et al. states that individuals affected by XLH are satisfied with the perceived quality of dental treatment itself, but indicate dissatisfaction with German healthcare support [14]. Also recent studies show that dentists often have too little experience with rare diseases, which makes it difficult for them to treat these patients adequately [38, 39].

It is essential to consider what adequate dental treatment for XLH patients should look like. The priority should be to increase public awareness about rare diseases. Medical professionals should be trained to recognize signals of such a disease. This can also be deduced from a study by Nguyen et al., in which it was shown that OHRQoL is improved when patients are seen and treated by professionals with the appropriate knowledge [40]. Another critical point is to initiate adequate treatment. This can be either the initiation of an adequate treatment or referral to a specialist. Especially in hypophosphatemia, therapists are faced with a difficult task, as there are few clinically visible symptoms. This makes it even more important to pay attention to patient reports. Even with clinically physiological findings at first glance, patients with XLH should be diagnosed further in order to identify and treat possible carious lesions at an early stage and thus prevent apical infections.

## Limitations

The major limitation of this study was the small number of study participants and the potential for sampling bias. Since there is no central registry for rare diseases such as XLH in Germany, only people affected by XLH who were members of a support group or who sought consultation at the University Hospital of Münster were included in this study. However, participants were affected by a rare disease which should be considered.

Another limitation of this study is the significant gender imbalance. Of the 26 participants, only five were men. A recent study by Bohner et al. [36] found no correlation between OHRQoL and sex in patients with rare diseases. Therefore, gender imbalance should not affect the outcome of our study. Further studies with more participants are needed. However, this is difficult to achieve, especially in studies of rare diseases.

The study design should also be extended in follow-up studies. Although our study design showed that both objective oral health and subjective OHRQoL were reduced in our cohort, the results should be confirmed by radiological findings and control groups. Radiographic findings could be used to better capture the apical changes relevant to XLH, which is only partially possible with the PhOX score we used.

## Conclusions

The results of this study show that people with XLH have impaired OHRQoL and poorer measured oral health compared to the general population in Germany using the PhOX score. Larger case–control studies should be conducted in the future to gain a more accurate picture of the disease and the individual factors that negatively influence oral health. On the one hand, XLH patients need to maintain good oral hygiene, but on the other hand they also need regular dental check-ups and prophylactic measures to detect oral manifestations soon as possible.

## Data Availability

All data generated or analyzed during this study are included in this published article.

## Abbreviations

XLH: X-linked hypophosphatemia
OHRQoL: Oral Health-related Quality of Life
OHIP14G: Oral Health Impact Profile 14 (German translation)
PHEX: Phosphate-regulating endopeptidase
PhOX: Physical Oralh Health Index
IQR: Interquartile range
CI: Confidence interval
